# PAciFy Cough—a multicentre, double-blind, placebo-controlled, crossover trial of morphine sulphate for the treatment of pulmonary Fibrosis Cough

**DOI:** 10.1186/s13063-022-06068-4

**Published:** 2022-03-02

**Authors:** Zhe Wu, Winston Banya, Nazia Chaudhuri, Ira Jakupovic, Toby M. Maher, Brijesh Patel, Lisa G. Spencer, Muhunthan Thillai, Alex West, John Westoby, Marlies Wijsenbeek, Jaclyn Smith, Philip L. Molyneaux

**Affiliations:** 1 Royal Brompton Hospital, Guy’s and St Thomas’ NHS Foundation Trust, London, UK; 2 National Heart and Lung Institute, Imperial College London, London, UK; 3 Manchester University NHS Foundation Trust, Manchester, UK; 4 Keck School of Medicine, University of Southern California, Lon Angeles, USA; 5 Liverpool Interstitial Lung Disease Service, Aintree University Hospital, Liverpool University Hospitals NHS Foundation Trust, Liverpool, UK; 6 Royal Papworth Hospital; Department of Medicine, University of Cambridge, Cambridge, UK; 7 Guy’s and St Thomas’ NHS Foundation Trust, London, UK; 8 Royal Brompton Hospital, Guy’s and St Thomas’ NHS Foundation Trust; National Heart and Lung Institute, Imperial College London, London, UK; 9 Centre for Interstitial Lung Disease and Sarcoidosis, Erasmus University Medical Centre Rotterdam, Rotterdam, The Netherlands; 10 Division of Infection, Immunity and Respiratory Medicine, Manchester Academic Health Science Centre, University of Manchester, Manchester, UK

**Keywords:** Morphine, Cough, Idiopathic pulmonary fibrosis, Interstitial lung disease, Quality of life

## Abstract

**Background:**

Idiopathic pulmonary fibrosis (IPF) is a progressive disease that leads to lung scarring. Cough is reported by 85% of patients with IPF and can be a distressing symptom with a significant impact on patients’ quality of life. There are no proven effective therapies for IPF-related cough. Whilst morphine is frequently used as a palliative agent for breathlessness in IPF, its effects on cough have never been tested. PAciFy Cough is a multicenter, double-blind, placebo-controlled, crossover trial of morphine sulphate for the treatment of cough in IPF.

**Methods:**

We will recruit 44 subjects with IPF prospectively from three interstitial lung disease units in the UK, namely the Royal Brompton Hospital, Manchester University NHS Foundation Trust (MFT) and Aintree University Hospital NHS Foundation Trust. Patients will be randomised (1:1) to either placebo twice daily or morphine sulphate 5 mg twice daily for 14 days. They will then crossover after a 7-day washout period. The primary endpoint is the percent change in daytime cough frequency (coughs per hour) from baseline as assessed by objective cough monitoring at day 14 of treatment.

**Discussion:**

This multicentre, randomised trial will assess the effect of opioids on cough counts and cough associated quality of life in IPF subjects. If proven to be an effective intervention, it represents a readily available treatment for patients.

**Trial registration:**

The study was approved by the UK Medicines and Healthcare Regulatory Agency (Ref: CTA 21268/0224/001-0001 – EUDRACT 2019-003571-19 – Protocol Number RBH2019/001) on 08 April 2020, in compliance with the European Clinical Trials Directive and the Medicines for Human Use (Clinical Trials) Regulations 2004 and its subsequent amendments. The study was provided with ethical approval by the London Brent Research Ethics Committee (Ref: 20/LO/0368) on 21 May 2020 and is registered with clinicaltrials.gov (NCT04429516) on 12 June 2020, available at https://clinicaltrials.gov/ct2/show/NCT04429516

**Supplementary Information:**

The online version contains supplementary material available at 10.1186/s13063-022-06068-4.

## Administrative information

Note: the numbers in curly brackets in this protocol refer to SPIRIT checklist item numbers. The order of the items has been modified to group similar items (see http://www.equator-network.org/reporting-guidelines/spirit-2013-statement-defining-standard-protocol-items-for-clinical-trials/).

**Table:** 

Title {1}	PAciFy Cough – A multicentre, double blind, placebo controlled, crossover trial of morphine sulfate for the treatment of PulmonAry Fibrosis Cough
Trial registration {2a and 2b}.	EudraCT number: 2019-003571-19
Protocol version {3}	8^th^ March 2021, version 4.1
Funding {4}	This study is fully funded by the Moulton Charitable Foundation.
Author details {5a}	Zhe Wu – Royal Brompton Hospital, Guy’s and St Thomas’ NHS Foundation Trust; National Heart and Lung Institute, Imperial College London Winston Banya – Royal Brompton Hospital, Guy’s and St Thomas’ NHS Foundation Trust Nazia Chaudhuri – Manchester University NHS Foundation Trust Ira Jakupovic – Royal Brompton and Harefield Hospitals, Guy’s and St Thomas’ NHS Foundation Trust Toby M Maher – Keck School of Medicine, University of Southern California Brijesh Patel – Royal Brompton Hospital, Guy’s and St Thomas’ NHS Foundation Trust Lisa Spencer – Liverpool Interstitial Lung Disease Service, Aintree University Hospital, Liverpool University Hospitals NHS Foundation Trust Muhunthan Thillai – Royal Papworth Hospital; Department of Medicine, University of Cambridge Alex West – Guy’s and St Thomas’ NHS Foundation Trust John Westoby – lay member Marlies Wijsenbeek – Centre for Interstitial Lung Disease and Sarcoidosis, Erasmus University Medical Centre Rotterdam Jaclyn Smith - Division of Infection, Immunity and Respiratory Medicine, Manchester Academic Health Science Centre, University of Manchester Philip L Molyneaux - National Heart and Lung Institute, Imperial College London & Royal Brompton Hospital, Guy’s and St Thomas’ NHS Foundation Trust
Name and contact information for the trial sponsor {5b}	Royal Brompton and Harefield Hospitals (RBHH) Ira Jakupovic, Research Governance and Regulatory Compliance Manager Guy’s and St Thomas’ NHS Foundation Trust (GSTFT) Royal Brompton and Harefield Hospitals (RBHH) Research Office, Chelsea Wing, Level 2, Sydney Street, London, SW3 6NP
Role of sponsor {5c}	The study sponsor has overseen the design of the study and will have oversight of the trial. The sponsor has ensured that the trial protocol, Patient Information Sheet (PIS), Informed Consent Form (ICF), GP letter and submitted supporting documents have been approved by the MHRA and a main Research Ethics Committee (REC), prior to any patient recruitment taking place. This study will be conducted in compliance with the protocol approved by the REC and according to GCP standards and UK Clinical Trials Regulation. Data ownership rights will lie with the institution. Our expectation is that after data analysis, information from this study will be widely disseminated in the medical and scientific community.

## Introduction

### Background and rationale {6a}

Idiopathic pulmonary fibrosis (IPF) is a chronic, progressive, fibrotic lung disease of unknown cause [1]. It is irreversible and responsible for 1 in every 100 deaths each year in the UK. Despite the recent approval of two antifibrotic therapies the 5-year survival rate remains 25%, far worse than many common cancers [2]. Whilst current therapies and those in development are understandably targeted at slowing the relentless progression of the disease, none attempt to alleviate any of the significant symptoms suffered by patients with IPF [3]. The most common symptoms reported by patients are fatigue (95%), dyspnoea (88%) and cough (85%) [4]. Cough can be a distressing symptom with significant physical, social and psychological consequences, and has been shown to predict disease progression [5]. The majority of patients with IPF report cough at some point during their disease [6], and it has been associated with a marked impairment in quality of life [7, 8].

The pathogenesis of cough in IPF is poorly understood. Patients with IPF have been shown to have a more sensitive cough reflex compared to healthy controls—both in response to inhaled challenge agents and to mechanical stimuli [9]. There is also a suggestion that genetic polymorphisms which contribute to the risk of developing IPF may also be implicated in IPF-related cough [10]. The lack of pathogenic clarity has limited the therapeutic options available to patients and clinicians, and cough in IPF remains one of the most challenging symptoms to address. Thalidomide has been shown to be beneficial in a randomised control trial; however, its side effects profile renders it practically useless as only 20% of patients are able to tolerate it [11]. Pirfenidone, one of the novel antifibrotic agents, has shown some promise in a recent trial [12] as has a nebulised form of sodium cromoglicate [13]. The reduction in objective cough frequency demonstrated by the currently available drugs to treat cough in IPF is in the order of 30%. However, this reduction has not consistently been associated with patient reports of improvements in coughing, questioning the clinical benefit afforded. There is therefore a clear unmet need to help reduce cough and improve the quality of life for patients with IPF.

Opiates have long been advocated for the suppression of cough [14]. Morphine is thought to depress the cough reflex, acting directly on the neural pathways in the brain. Antitussive effects occur with doses lower than those usually required for analgesia. In patients with refractory chronic cough, 5–10 mg of controlled-release morphine sulphate every 12 h has been shown to cause significant suppression of cough, reducing objective cough frequency by over 70% compared to placebo in clinical responders [14]. Whilst morphine is frequently used as a palliative agent for dyspnoea in IPF, its effects on cough have never been tested [15].

The only randomised control trial evaluating opioids in chronic cough was conducted by Morice and colleagues [14] in 27 patients, where a starting dose of 5 mg twice daily slow-release morphine (MST) was shown to be effective in reducing diary recorded cough scores. The study ran an open-label extension period, allowing patients to increase the dose of MST to 10 mg twice daily according to patient choice. Interestingly, there was no significant difference in cough severity or Leicester Cough Questionnaire (LCQ) score between those that took 5 mg and 10 mg twice daily. Furthermore, despite the side effects of constipation and drowsiness, all patients completed the study. This highlights the tolerability of low-dose MST.

A large longitudinal cohort study in Sweden with over 1600 oxygen-dependent ILD patients revealed that opiates were used in 15% of patients and was not associated with either increased mortality or hospitalisation [16]. This was true for both low and high dose (equivalent of 30 mg daily oral morphine or higher) therapy. This study confirms the safety of opioids in ILD patients, even in those who have more severe disease.

We present the protocol of a multi-centre, randomised, double-blind, placebo-controlled, crossover trial investigating the effect of low-dose morphine on cough in patients with IPF. If this study shows a favourable outcome of morphine as a treatment for IPF-related cough, this could be rapidly translatable into routine clinical practice. The data generated would also provide the information needed to plan future studies, to explore optimal dosing as well as adjunctive antitussive therapies in IPF.

### Objectives {7}

The primary objective of this study is to establish whether, compared with placebo, low-dose (5 mg twice daily) controlled release morphine sulphate (MST) will reduce the number of coughs recorded during a 24-h period in patients with IPF at 14 days of treatment.

The secondary objectives include:

- Evaluating the within subject differences in self-reported (LCQ, cough VAS) and objective cough frequency with morphine compared to placebo;
- Assessing the changes in response to morphine therapy on health-related quality of life, anxiety and dyspnoea with a range of quality of life questionnaires (L-IPF, HADS, K-BILD, D12)
- Assessing the relationship between physical activity (as measured by step counts every 15 min via a Fitbit device) and objective cough counts;
- Evaluating a range of exploratory blood biomarkers for cough and response to morphine.

### Trial design {8}

**Fig. 1:**
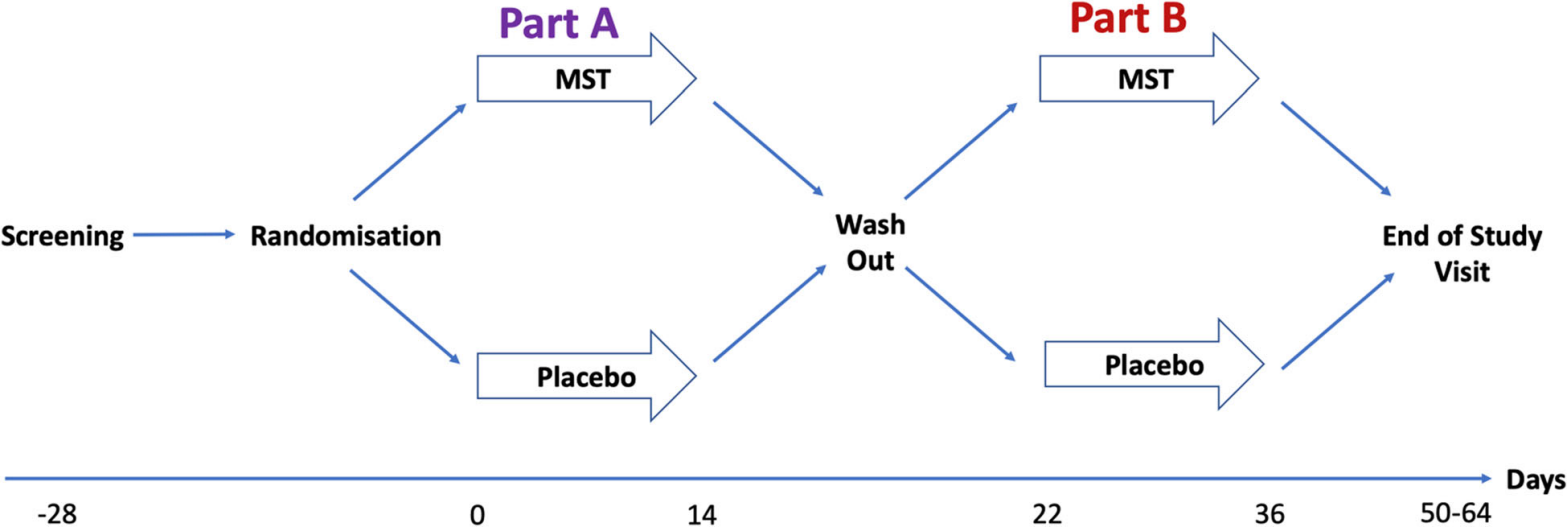
A randomised, double-blind, placebo-controlled, two-way crossover study investigating the effect of MST on cough in IPF

The PAciFy Cough study is a UK, multicentre, randomised, double-blind, placebo-controlled, two-way crossover trial of controlled release morphine sulphate (MST) in subjects with idiopathic pulmonary fibrosis (IPF). Patients will be randomised (1:1) to either placebo twice daily or MST 5 mg twice daily for 14 days. Patients will then crossover after a 7-day washout period. Those that were randomised to placebo will be given MST 5 mg twice daily and those that were randomised to MST will take placebo for 14 days. The study design is outlined in Fig. 1.

## Methods: participants, interventions and outcomes

### Study setting {9}

PAciFy Cough will recruit 44 subjects with IPF prospectively from three interstitial lung disease units in the UK, namely the Royal Brompton Hospital, Manchester University NHS Foundation Trust (MFT) and Aintree University Hospital NHS Foundation Trust.

### Eligibility criteria {10}

The inclusion criteria are:

- Self-reported cough (> 8 weeks) with cough VAS ≥ 30/100
- A diagnosis of IPF within 5 years prior to the screening visit, as per applicable ATS/ERS/JRS/ALAT guidelines, in line with hospital records
- Male or Female between the age of 40 and 90 years
- Male participants: a male participant must agree to use contraception as detailed in Appendix 2 of the protocol during the study and for at least 90 days after the follow-up visit, and refrain from donating sperm during this period
- Female participants: A female participant is eligible to participate if she is not pregnant, not breastfeeding, and not a woman of childbearing potential (WOCBP) as defined in Appendix 2 of the protocol
- Meeting all of the following criteria during the screening period: FVC ≥ 45% predicted of normal, forced expiratory volume in 1 s (FEV1)/FVC ≥ 0.7, DLCO corrected for Hb ≥ 30% predicted of normal. Lung function performed within 12 months of screening is acceptable
- The extent of fibrotic changes is greater than the extent of emphysema on the most recent HRCT scan (investigator determined within 24 months of the study screening visit)
- Able to provide written informed consent

Patients will be excluded if any of the following are met:

- Treatment with immunosuppressive therapy or antibiotics within last 4 weeks of screening visit. A stable dose of corticosteroids equivalent to prednisolone of 10 mg per day or less, if used for an indication other than pulmonary disease will be permitted
- Current smoker
- History of alcohol and drug(s) addiction
- Acute IPF exacerbation within 6 months prior to screening and/or during the screening period
- Concurrent use of pirfenidone or nintedanib, unless receiving a stable dose for at least 8 weeks prior to screening
- Use of ACE inhibitors
- Patients with co-existent conditions known to be associated with the development of fibrotic lung disease. This includes connective tissue disease, suspected drug-induced lung disease, asbestosis or other asbestos-related disease (pleural plaques, mesothelioma) and granulomatous disease including sarcoidosis. Patients with an auto-immune profile considered diagnostic for a specific connective tissue disease will be excluded, even in the absence of systemic symptoms. Non-specific rises in auto antibodies, e.g. rheumatoid factor, anti-nuclear antibody, etc., will not be used to exclude individuals from the study
- Significant other organ co-morbidity including hepatic or renal impairment and pulmonary hypertension (investigator determined)
- Significant coronary artery disease (myocardial infarction within 6 months or ongoing unstable angina within 4 weeks of screening visit) or congestive cardiac failure based on clinical examination
- Patients at significant risk for side effects, intolerance or allergy to morphine
- Pregnant patients, or women of child-bearing potential, not using a reliable contraceptive method. A urine pregnancy test will be performed in females of child-bearing potential at the initial study visit
- Predicted life expectancy < 6 months
- Use of long-term oxygen therapy. Use of ambulatory oxygen will be permitted
- Current or use of opiates within 14 days of the screening visit
- Unable to provide informed written consent

Drop out criteria:

- Significant intolerance or allergic reaction to IMP
- Patient withdrawal of consent

### Who will take informed consent? {26a}

Informed consent (see additional file 1—ICF) will be obtained by the chief investigator (CI), principal investigator (PI) and/or a nominated deputy as recorded on Sponsor’s Delegation of Responsibilities Log. Only those members of the study team who have clinical responsibility for the care of patients under the care of the general medical service will be permitted to undertake informed consent. All individuals taking informed consent will have received training in Good Clinical Practice (GCP). Consent to enter this study will be obtained after a full account has been provided of its nature, purpose, risks, burdens and potential benefits, and the patient has had the opportunity to deliberate.

Patients with an established diagnosis of IPF will be approached at a clinic, the study will be explained, and a participant information sheet (PIS) will be given to interested subjects. Patients will also be identified from an existing database of IPF patients specific to each site, and an invitation letter and information sheet describing the study will be sent by post. This will be followed by a telephone conversation a minimum of 24 h later to establish interest and answer any questions before booking in for visit 1. Patients will be provided with a full explanation of the study at visit 1 and be given the opportunity to ask questions before providing written consent.

### Additional consent provisions for collection and use of participant data and biological specimens {26b}

A separate blood samples consent form for the collection of blood for biomarkers, RNA and DNA will be used (see additional file 2—blood ICF). If a subject does not wish to have these samples collected, this will not affect their participation in the trial.

### Interventions

#### Explanation for the choice of comparators {6b}

Morphine is thought to depress the cough reflex by direct effect on the cough centre in the brain. Antitussive effects occur with doses lower than those usually required for analgesia. In this study, patients will take 5 mg of MST every 12 h. This dose has been shown to cause significant suppression of cough in patients with refractory chronic cough [14]. Indeed, in the study by Morice and colleagues, an increased dose of MST from 5 mg to 10 mg twice a day resulted in increased drowsiness, without a significant improvement in reported cough severity.

#### Intervention description {11a}

Subjects will be randomised sequentially to a sequence group defining the order in which active drug and placebo are given, according to a computer-generated schedule (Sealed Envelope EDC). Access to the database at each participating centre will be restricted to authorised study staff. Patients will be randomised (in a 1:1 double-blind fashion) to receive either morphine sulphate in part A followed by placebo in part B or placebo in part A followed by morphine sulphate in part B (see Fig. 1). Part A (day 0 to 14) and part B (day 22 to 36) will each be 14 days of treatment with a 7-day washout period.

There will be a fixed dose of MST 5 mg twice daily, and there will be no dosage adjustments. Patients will be instructed to take one capsule (placebo or morphine sulphate prolonged release 5 mg) orally twice daily according to the prescribed dosing schedule. The researcher will check patients’ compliance at the end of each treatment period. To assess compliance, the number of tablets returned to the hospital pharmacy will be compared to the number distributed and assigned dose level.

#### Criteria for discontinuing or modifying allocated interventions {11b}

There will be a fixed dose of MST 5 mg twice daily, and no dosage adjustments will be permitted. Patients may decide to withdraw early from the study, or the research physician may feel that it is in the best interests of the patient to terminate their involvement in the study prior to completion for safety reasons. Patients who wish to withdraw their consent do not have to give a reason to do so. Early withdrawal will be clearly documented in the case report form (CRF) and the hospital case notes. All unused medications will be returned.

Patients will be withdrawn from the study if they develop a condition that would compromise their safety, and the decision is made by an Investigator. Patients who withdraw from the study will not be replaced. If the sponsor decides to terminate the study, the patients will be informed and the reason for termination will be documented in the CRF.

#### Strategies to improve adherence to interventions {11c}

The researcher will check patients’ compliance at the end of each treatment period. To assess compliance, the number of tablets returned to the hospital pharmacy will be compared to the number distributed and assigned dose level.

#### Relevant concomitant care permitted or prohibited during the trial {11d}

Patients will be permitted to use laxatives for side effect of constipation, and anti-emetics for nausea. The use of these medications will be documented in the clinical notes. Patients will be permitted to take concomitant anti-tussive medications and inhalers if they have been on a stable dose of these treatments for at least 4 weeks prior to the screening visit. Dose titrations of these medications will not be permitted. Between day 0 and day 35, patients will not be permitted to receive additional opiates (e.g. oramorph) or to use ACE inhibitors.

#### Provisions for post-trial care {30}

Patients will be assessed on a case by case basis across all participating sites and will be given the option to remain on the study drug after completion of the trial if there appears to be a benefit to them.

#### Outcomes {12}

Primary outcome:

The primary efficacy endpoint is the percent change in daytime cough frequency (coughs per hour) from baseline as assessed by objective digital cough monitoring at day 14 of treatment [17].

Secondary outcomes:

- Change from baseline in health-related quality of life scores (L-IPF, HADS, K-BILD)
- Change from baseline in self-reported cough (LCQ and VAS)
- Change from baseline in dyspnoea (D-12)
- Change from baseline in global impression of change in quality of life, cough and breathlessness
- The proportion of responders with a minimum of 20% decrease from baseline at the end of treatment in 24-h average cough count

Quality of life, self-reported cough and breathlessness will be assessed by self-administered questionnaires. These are completed at baseline and repeated at all follow-up visits and the end of study visit. The instruments used will be the Leicester Cough Questionnaire (LCQ), Cough severity VAS, Dyspnoea-12 (D-12), Living with IPF (L-IPF), Kings Brief ILD and the Hospital Anxiety and Depression Scale (HADS) questionnaire. A global impression of change in quality of life, cough and breathlessness will also be recorded. The minimal clinically important difference (MCID) for total KBILD score in IPF patients is 3.9, but the MCID for the other quality of life measures are yet to be established in IPF [18].

Exploratory outcomes:

- Change in candidate serum biomarkers of fibrosis following therapy
- Association of activity (as measured by step count) and cough frequency

#### Participant timeline {13}

Patients will be asked to attend the department for study visits on 3 occasions (days 0, 22 and 36). In addition to three [3] hospital visits, the research team will conduct two [2] remote study visits (days 14 and 50) to ensure safety of the study patients for the duration of the study. Figure 1 is a schematic diagram of the trial timeline.

#### Sample size {14}

Based on previous repeatability data in IPF patients studied over an 11-day period, a sample size of 40 subjects will have 90% power to detect a true difference in 24 h cough frequency on the natural log scale − 0.132 or 0.132 (equivalent to ~ 35% change) with a probability (power) of 0.9, assuming a within-subject standard deviation of 0.310 (natural log scale) and the standard significance level of 0.05. Allowing for a 10% drop out rate, we will aim to recruit 44 patients for the study. In a previous phase 2 study, Birring and colleagues demonstrated a 30% reduction in log transformed cough counts in IPF subjects compared to baseline [13]. Therefore, a 35% reduction in cough counts in this study is likely to be of clinical importance.

#### Recruitment {15}

Recruitment will be from three ILD units in the UK, as specified in the methods section.

#### Patient involvement in protocol design

An IPF support group at RBH consisting of patients, their families and carers was consulted on the initial and revised protocol design. They provided positive feedback, commenting on the value of having the option to continue taking morphine in the community following their participation in the trial. Following the emergence of the COVID pandemic, the support group were consulted again, who suggested a reduction in the number of onsite visits, and thus the revised protocol was amended to reflect this. Finally, we have a lay member on the trial steering committee.

## Assignment of interventions: allocation

### Sequence generation {16a}

Subjects will be randomised sequentially to a sequence group defining the order in which active drug and placebo are given, according to a computer-generated schedule (Sealed Envelope EDC). Block randomisation of size 4 or 6 will be undertaken, and a unique kit code will be assigned to each treatment kit (one bottle containing 30 capsules of either morphine or placebo). Access to the Sealed Envelope system at each participating centre will be restricted to authorised study staff. The code list, detailing specific kit codes to the corresponding treatment arm, will be blinded from the investigator. Subject recruitment will be performed by the investigator and research nurses at each site, who are trained according to the trial protocol.

### Concealment mechanism {16b}

See text in the “Sequence generation {16a}” section.

### Implementation {16c}

See text in the “Sequence generation {16a}” section.

## Assignment of interventions: blinding

### Who will be blinded {17a}

This will be a double-blind study with both the patient and investigator blinded to study treatment. The investigational medicinal product (IMP) is an over-encapsulated morphine sulphate prolonged-release 5 mg tablet. The IMP and placebo capsules are both coloured Swedish orange to maintain blinding. One bottle containing 30 capsules of either morphine or placebo will be provided for each treatment period. Each bottle is assigned a unique kit code.

### Procedure for unblinding if needed {17b}

The investigator or treating physician may un-blind a patient’s treatment assignment in the case of an emergency, when knowledge of the study treatment is essential for the appropriate clinical management or welfare of the patient. The investigator will make the decision to un-blind and have 24-h access to un-blinding the treatment assignment via the electronic database system (Sealed Envelope EDC). If the Sealed Envelope EDC system is not accessible for technical reasons (e.g. electronic failure of the database), a manual back up system is also available. A master randomisation list will be provided to the Sponsor (RBHH) pharmacy department. If the Sealed Envelope EDC system is not accessible for technical reasons, then the investigator will contact RBHH hospital pharmacist (and/or an on-call pharmacist outside of working hours) via RBHH switchboard for the manual un-blinding of the treatment assignment. The investigator must notify the Sponsor as soon as possible. The date and reason for the unblinding must be recorded in the appropriate data collection tool, eCRF. A patient will be withdrawn if their treatment code is un-blinded by the investigator or treating physician. The primary reason for discontinuation (the event or condition which led to the unblinding) will be recorded in the eCRF.

### Data collection and management

#### Plans for assessment and collection of outcomes {18a}

Measurement of cough frequency will be performed using objective digital cough monitoring. The digital cough monitor (VitaloJAK) consists of a portable sound recording device, which is worn in a pouch or pocket. The small microphone is clipped onto the subject’s collar or lapel, as close as possible to the anterior neck. A continuous sound recording is made for up to 24 h. The digital recording is then processed through accompanying computer software, which automatically registers sound patterns typical for cough. Software output provides the total number of cough events over the entire time period of recording and average hourly cough frequency. A recording will be performed before and after each treatment period.

Quality of life questionnaires can either be completed electronically on-site or sent to participants’ email addresses. These are completed at baseline and repeated at follow up visits. Routine safety blood tests including full blood count, renal and liver function tests will be undertaken in the clinical laboratories at local sites according to local policies and procedures. Copies of local reference ranges will be collected.

#### Plans to promote participant retention and complete follow-up {18b}

In light of the COVID-19 pandemic, the protocol was modified to minimise the number of face to face visits. This was received favourably by patient support groups. Patients will be pre-screened with a telephone call to ensure that the inclusion and exclusion criteria are met before their first visit.

#### Data management {19}

Data will be collected on an eCRF system. The Sealed Envelope EDC has been designed following the requirements of the clinical trial protocol and complies with regulatory requirements. Local personnel will be trained on the Sealed Envelope EDC system. Access will be restricted to site personnel, trial managers, trial monitors and the data management team. Personnel will have individual log-on and passwords. It will be the investigator’s responsibility to ensure the accuracy of all data entered and recorded in the eCRFs. Trial monitors will check the accuracy of the eCRF data against source documents. Source documents are original documents and records from which participants’ data are obtained. These also include, but are not limited to, hospital records, clinical and office charts, laboratory and pharmacy records, diaries, microfiches, radiographs, correspondence and paper CRF entries.

All data will initially be entered legibly in black ink with a ball-point pen in the paper CRF (study worksheets). If the investigator makes an error, it will be crossed through with a single line in such a way to ensure that the original entry can still be read. The correct entry will then be clearly inserted. The amendment will be initialled and dated by the person making the correction immediately. Overwriting or use of correction fluid will not be permitted. It is the investigator’s responsibility to ensure the accuracy of all data entered and recorded in the paper and electronic CRFs. The Delegation of Responsibilities Log will identify all trial personnel responsible for data collection, entry, handling and managing the database.

#### Confidentiality {27}

All data will be handled in accordance with the Data Protection Act 2018. The CRFs will not bear the subject’s name or other personally identifiable data. The subject’s initials, Date of Birth (DOB) and trial Identification Number (ID), will be used for identification. CRFs have been designed by the CI and the final version approved by the sponsor. A source document location log will be completed and filed in the investigator site file indicating what constitutes source data and where it will be located. All documents will be stored safely in confidential conditions. On all study-specific documents, other than the signed consent form, the participant will be referred to by the study participant number, not by name.

#### Plans for collection, laboratory evaluation and storage of biological specimens for genetic or molecular analysis in this trial/future use {33}

All biological samples for future research will be collected and handled according to a study-specific procedure, stored anonymously and labelled using a unique study number to permit accurate linkage to clinical data. Samples will be initially processed and stored at study sites in accordance with the study-specific procedure for handling PAciFy Cough biological samples, to facilitate transfer to the Biomedical Research Unit (BRU) Royal Brompton Hospital (RBH), Sydney Street, London, SW3 6NP. The exploratory analysis of potential serum biomarkers and blood transcriptomics may be undertaken at; the Royal Brompton Hospital (RBH), Imperial College London, a commercial research organisation or in collaborating academic institutions. Analysis of these samples may be undertaken after completion of the study and following assessment of the primary study outcome.

### Statistical methods

#### Statistical methods for primary and secondary outcomes {20a}

Summary statistics will be presented by treatment group. For continuous variables, unless otherwise stated, the number of available observations (*n*), mean, standard deviation, median and range will be provided. For categorical variables, the number and percentage in each category will be displayed. The primary efficacy endpoint is the change from baseline in log-transformed 24-h average cough count at the end of treatment. The primary analysis will be conducted using a linear mixed model for repeated measures (MMRM) adjusting for baseline measures and assessing any influence of treatment, centre, sequence or period. In the primary analysis model, all available data from each subject will be included. Secondary efficacy endpoints will be summarised using descriptive statistics. For continuous endpoints (e.g. change from baseline), summaries will include mean, median, standard deviation, minimum and maximum. For discrete variables (e.g. frequency), summaries will include number of instances and percentage of total instances for that category or time period. Quantitative secondary endpoints will be analysed using analysis of covariance (ANCOVA) models and proportion endpoints will be analysed using logistic regression models. All secondary analyses will be conducted using two-sided tests at the alpha = 0.05 level of significance.

#### Interim analyses {21b}

No formal interim analysis is planned.

#### Methods for additional analyses (e.g. subgroup analyses) {20b}

Not applicable.

#### Methods in analysis to handle protocol non-adherence and any statistical methods to handle missing data {20c}

To maximise completeness of data, patients will be called by a member of the trial team after each visit to remind them to return the cough monitor in a pre-paid envelope. During the telephone call, the participant will also be reminded to take the trial medication and complete the online quality of life questionnaires. Automated email reminders to complete the survey will be sent daily for three consecutive days after the visit date.

#### Plans to give access to the full protocol, participant level-data and statistical code {31c}

Details of the trial including study design, eligibility criteria and outcome measures are available to the public on clinicaltrials.gov (NCT04429516).

### Oversight and monitoring

#### Composition of the coordinating centre and trial steering committee {5d}

An independent trial steering committee (TSC) will be established to oversee the conduct of the study. It is anticipated that the TSC will comprise the lead investigators, an independent chair, two additional independent members, at least one of whom will be a patient/public representative. The TSC will develop terms of reference outlining their responsibilities and operational details. The TSC will meet after 15 patients have completed the study and as required during the trial.

#### Composition of the data monitoring committee, its role and reporting structure {21a}

There will not be a data monitoring committee (DMC).

#### Adverse event reporting and harms {22}

The checking for the occurrence of adverse events (AEs) and clinical endpoints will begin from randomisation and will continue for the individual patient until they complete their follow-up at 48 weeks. At each study visit, the investigator or designee will assess safety and will specifically review the clinical history and investigation findings with regard to the occurrence of adverse or serious adverse events (SAEs). Details of adverse and clinical events will be captured on the trial eCRF. All SAEs will be recorded in the hospital notes and the CRF, and the sponsor’s SAE Recording Log. The SAE Log will be sent to sponsor on request and every 2 months. All SAEs will be reported to the sponsor via the Research Office (RO) dedicated mailbox on an SAE form unless otherwise stated in the protocol. The chief or principal investigator will complete the sponsor’s SAE form and the form will be faxed to the RO within 24 h of the investigator becoming aware of the event. The chief or principal investigator will respond to any SAE queries raised by the sponsor as soon as possible.

Expected adverse reactions are those which are specified in ‘Undesirable Effects’ of the SmPC of morphine sulphate. Investigators must complete the appropriate SAE form on the eCRF and an automatic email notification will be sent to the trial manager, CI and sponsor.

Suspected unexpected serious adverse reaction (SUSAR) is an adverse reaction that is classed as both serious and unexpected. The trial manager will ensure the SUSAR report is un-blinded and is reviewed by the CI or designee within 2 days and adjudicate whether the event constitutes a SUSAR. The trial manager will ensure that fatal or life-threatening SUSARs are reported to the MHRA and the main REC as soon as possible, but no later than 7 calendar days after the receipt of the eSAE report. Any additional information will be reported within 8 days of sending the first report. The trial manager must report all other SUSARs and safety issues to the MHRA and main REC, as soon as possible but no later than 15 calendar days after the sponsor has first knowledge of the minimum criteria for expediting reporting.

#### Frequency and plans for auditing trial conduct {23}

The trial will be monitored according to the monitoring plan agreed and written by the sponsor, based on the internal risk assessment procedure. Where appropriate the CI will be asked to complete a copy of the sponsor’s self-monitoring template. It is the responsibility of the CI to ensure this is completed and submitted to the RO on request. It is the responsibility of the RO to determine the monitoring risk assessment and explain the rationale. It is the RO’s responsibility to ensure that any findings identified in a PI’s monitoring report are actioned in a timely manner and any violations of GCP or the protocol reported to the RO immediately. Any urgent safety measures at either the CI or a PI site must be reported by that site Investigator within 3 days, as per UK Regulations.

#### Plans for communicating important protocol amendments to relevant parties (e.g. trial participants, ethical committees) {25}

The trial protocol, patient information sheet (PIS), informed consent form (ICF), GP letter and submitted supporting documents have been approved by the MHRA and the Brent Research Ethics Committee (REC), before patient recruitment. All subsequent substantial protocol amendments will be documented and submitted for ethical and regulatory approval prior to implementation. Before site(s) can enrol patients into the trial, the principal investigator must apply for site-specific assessment from the Trust Research & Development (R&D) and be granted written NHS R&D approval. It is the responsibility of the principal investigator at each site to ensure that all subsequent amendments gain the necessary approval.

#### Dissemination plans {31a}

We expect that after data analysis, information from this study will be widely disseminated in the medical and scientific community. This will be achieved through a series of peer-reviewed publications and meeting abstracts at local, national and international events.

## Discussion

Cough in IPF remains an unmet clinical need, with no proven treatments. This multicentre, randomised trial will assess the effect of opioids on cough counts and cough associated quality of life in IPF subjects. The amended protocol takes into consideration the effects of the COVID-19 pandemic, with two of the five visits conducted remotely, whilst maintaining adequate safety monitoring and allowing data collection for all study objectives. If proven to be an effective intervention, this study may lead to further mechanistic studies for IPF and represents a readily available treatment for patients.

## Trial status

The current protocol attached is version 4.1, dated 8 March 2021. Recruitment began in December 2020, and the trial is currently recruiting in the UK. It is expected that recruitment will be completed by August 2022.

## Supplementary Information

**Additional file 1:.** Blood samples consent form

**Additional file 2:.** Informed consent form

## Abbreviations

AE: Adverse event
BRU: Biomedical Research Unit
CI: Chief investigator
CRF: Case report form
D12: Dyspnoea 12 Questionnaire
DMC: Data monitoring committee
eCRF: Electronic case report form
GCP: Good Clinical Practice
HADS: Hospital Anxiety and Depression Scale
ICF: Informed consent form
ILD: Interstitial lung disease
IMP: Investigational medicinal product
IPF: Idiopathic pulmonary fibrosis
K-BILD: King’s Brief ILD Questionnaire
LCQ: Leicester Cough Questionnaire
L-IPF: Living with IPF Questionnaire
MMRM: Mixed model for repeated measures
MST: Morphine sulphate
PI: Principal investigator
PIS: Patient information sheet
RBH: Royal Brompton Hospital
RBHH: Royal Brompton and Harefield Hospitals
REC: Research ethics committee
RO: Research office
SAE: Serious adverse event
SUSAR: Suspected unexpected serious adverse reaction
TSC: Trial steering committee
VAS: Visual analogue scale
