# Supplementary material for: PAciFy Cough—a multicentre, double-blind, placebo-controlled, crossover trial of morphine sulphate for the treatment of pulmonary Fibrosis Cough
Source: Trials. 2022 Mar 2;23:184. doi: 10.1186/s13063-022-06068-4 (PMC8889046; doi:10.1186/s13063-022-06068-4)
Supplement: Supplementary file 1 — Additional file 1:. Blood samples consent form [file 13063_2022_6068_MOESM1_ESM.docx]

**INFORMED CONSENT FORM**

**Project Title:** A multicenter, double blind, placebo controlled, crossover trial of morphine sulphate for the treatment of PulmonAry Fibrosis Cough (PAciFy Cough)

**Name of Researcher:**

|  | **Please Initial in box** |
| --- | --- |
| 1. I confirm that I have read and understand the information sheet dated (**Version 4.3, 08^th^ March 2021**) for the above study and have had the opportunity to consider the information, ask questions and have these answered satisfactorily. | **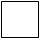** |
| 1. I understand that my participation is voluntary and that I am free to withdraw at any time, without giving any reason, without my medical care or legal rights being affected. | **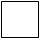** |
| 1. I understand that relevant sections of any of my medical notes and data collected during the study may be looked at by responsible individuals from Imperial College or from regulatory authorities where it is relevant to my taking part in research.   I give permission for these individuals to have access to my records. | **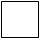** |
| 1. I agree with the publication of the results of this study in a medical journal   (all data will be published anonymously). | **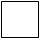** |
| 1. I consent to 24-hour cough recording. I have been informed that the monitor records   all sound, not just my cough. I understand that disclosure of information to the appropriate authority may be necessary should the monitor record anything which may put me or another at danger or risk. | **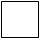** |
| 1. I consent to the upload of my cough recordings to Vitalograph portal to ensure its safe storage of in RaDAR under the terms of this study for a maximum of 20 years. | **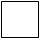** |
| 1. I understand that anonymized data and recordings may be made available for further research, subject to application process and approved by the RaDAR Management Team. I consent for my recordings to be used for future research: |  |
| 1. In the UK | **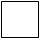** |
| 1. In the EEA. | **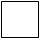** |
| 1. I agree for my GP to be informed about my participation in the study. |  |
| 1. I understand that my blood and urine samples, including my DNA samples, will be analyzed, anonymously, by researchers outside my local hospital, potentially including the EEA. I also understand that after the end of the study samples will be stored and may be used, anonymously, in future ethically approved research. | **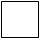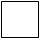** |
| 1. I agree to be contacted by phone following each study visit. | **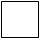** |
| 1. I agree to take part in the above study. | **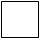** |

_______________________ ____________________ ____________________

Name of participant Date Signature

________________________ ____________________ ____________________

Name of person taking consent Date Signature
