# Supplementary material for: PAciFy Cough—a multicentre, double-blind, placebo-controlled, crossover trial of morphine sulphate for the treatment of pulmonary Fibrosis Cough
Source: Trials. 2022 Mar 2;23:184. doi: 10.1186/s13063-022-06068-4 (PMC8889046; doi:10.1186/s13063-022-06068-4)
Supplement: Supplementary file 2 — Additional file 2:. Informed consent form [file 13063_2022_6068_MOESM2_ESM.docx]

**BLOOD SAMPLES CONSENT FORM**

**Project Title:** A multicenter, double blind, placebo controlled, crossover trial of morphine sulphate for the treatment of PulmonAry Fibrosis Cough (PAciFy Cough)

**Name of Researcher:**

|  | **Please Initial in box** |
| --- | --- |
| 1. I give permission for my medical information to be stored on the Biomarker core lab and the Genetic core lab databases. I understand that this information will be passed on to researchers outside of the Trust in an anonymous form (*i.e.* without my name and address). ALL samples used for genetic research studies will be anonymised to the researcher. | **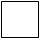** |
| 1. I understand my medical information may be published in an anonymous form with any research findings. | **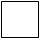** |
| 1. I understand that my participation is voluntary, and I am free to withdraw at any time, without giving any reason, without my medical care or legal rights being affected. | **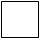** |
| 1. I understand that these samples will be treated as a gift or donation and I will not   receive any financial remuneration. | **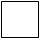** |
| 1. I give permission for my samples to be used for research in commercial organizations (such as a company manufacturing a drug) located: |  |
| 1. within the UK | **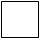** |
| b) to the EEA. | **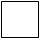** |
| 1. I give permission for my samples to be used for research in other hospitals or academic laboratories located: |  |
| 1. within the UK | **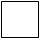** |
| b) to the EEA. | **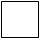** |
| 1. I understand that my samples may be used for genetic (DNA and/or RNA) studies and that this analysis may be carried out with other collaborative research institutions, such as the Sanger Institute. | **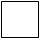** |

_______________________ _________________ _______________________

Name of participant Date Signature

_________________________ _________________ _______________________

Name of person taking consent Date Signature
